# The Depiction of Medical Errors in a Sample of Medical Television Shows

**DOI:** 10.7759/cureus.11994

**Published:** 2020-12-09

**Authors:** Molly Carney, Tonya S King, Anna Yumen, Carissa Harnish-Cruz, Renyta Scales, Robert P Olympia

**Affiliations:** 1 Pediatrics, Penn State College of Medicine, Hershey, USA; 2 Public Health Sciences, Penn State College of Medicine, Hershey, USA; 3 Emergency Medicine, Penn State College of Medicine, Hershey, USA; 4 Pediatric Emergency Medicine, Penn State College of Medicine, Hershey, USA

**Keywords:** medical error, adverse events, healthcare quality, medical education, continuing education

## Abstract

Background: Medical errors and adverse events may affect up to 7.5% of hospitalizations, although observational studies suggest the numbers could be even higher. Previous studies have shown that medical television (TV) shows may be a major driver when it comes to a patient’s medical knowledge and perspectives.

Methods: Six episodes from the first season of eight medical TV series were analyzed by four reviewers. Demographics of the healthcare provider responsible for the error, demographics of the victim, type of error, setting of error, level of disability, and reporting of the error were recorded. Data was compared with event rates from US hospitals.

Results: A total of 242 medical errors (average 6.4/hr) were included in the analysis. The healthcare provider responsible for the error was often an attending physician (55.8%), while victims were often White (73.6%), males (55.0%), aged 16-44 years (50.8%). Errors in diagnosis (28.9%) and operative errors (19.4%) were most common. Compared with data from US hospitals, TV series depicted more errors in diagnosis (p<0.001) and fewer operative errors (p<0.001). The most common levels of disability following medical errors were emotional trauma (37.6%) and temporary injury (30.2%). Emotional trauma was significantly overrepresented and temporary injuries were underrepresented (p<0.001). Error was not reported to the victim in 49.2% of events.

Conclusion: There were multiple discrepancies between errors depicted on TV and US hospital data. This may lead to viewer fear and anxiety that results in delays in seeking medical care and increased medicolegal cases. Healthcare systems should attempt to reduce the incidence of medical errors and adverse events by ensuring competencies of their providers, instituting methods of risk analysis and prevention, and training providers on methods of proper error disclosure.

## Introduction

Medical errors are responsible for up to 251,000 deaths in the United States each year, making it the third leading cause of death [1]. In addition to mortality, medical errors and negligence can lead to disability, delays in recovery or emotional trauma [2]. Data from US hospitals shows that adverse events occur in 3.2%-7.5% of hospitalizations, with the most common errors being operative (44.9%-49.3%) or drug-related (15.7%-19.3%) errors that result in temporary injury (16.5%-73.8%) or insignificant injury (10.8%-56.8%) [3-6]. However, observational studies have shown even higher rates of medical error [7]. Observers of a surgical unit in a Chicago teaching hospital found that up to 45.8% of patients experienced an event associated with an inappropriate decision. Of the 1,047 patients studied, 17.7% experienced a serious event but only 1.2% filed a claim, evidencing how even serious medical errors can go unreported [8].

Although this data speaks to the prevalence of medical error occurring in the US healthcare system, many healthcare consumers may be unaware of these statistics until it personally affects them. With the increasing popularity of medical television (TV) shows, viewers often get a dramatized look into the world of medicine. Cultivation theory argues that repeated exposure to events or ideas in the media can influence people’s beliefs about the real world [9]. For example, several studies have found that the general population lists TV as a primary source of information and education about resuscitation [10]. Unfortunately, medical TV shows are known to over-exaggerate medical and traumatic scenarios to capture the interest of their audience. Several studies examining medical TV shows have shown that the survival rates associated with cardiopulmonary resuscitation (CPR) events are often inaccurate, and favor improved short-term and long-term survival rates when compared with reports for in-hospital CPR [10-13]. Another example of over-exaggeration in medical TV shows is in the depiction of major disasters. In one study describing major disasters depicted in ED-associated medical TV shows, the authors demonstrated an average mortality rate 0.14% higher than the weighted mortality rate of 10 major US disasters [14].

To our knowledge, there have been no published studies examining the depiction of medical errors depicted in US medical TV shows. The objective of this study was to characterize the depiction of medical errors in a sample of eight medical TV shows and compare these characterizations with the US hospital data.

## Materials and methods

Data collection

Eight medical TV series released during 1994-2018 were selected for this study (Table 1). In order to represent medical TV series over several decades, four series released before 2005 (ER, Scrubs, House and Grey's Anatomy) and four series released after 2005 (The Resident, The Good Doctor, Code Black and Chicago Med) were selected. Series were selected based on high ratings and viewership at the time of release, as well as diversity of medical settings and genres (Table 1). Since each series had a different number of episodes during Season 1, the first and last three episodes of the first season of each series were independently reviewed by four medical student reviewers.

**Table 1:** Description of medical TV series

Medical Series Name	Network	Genre	Date Premiered	Episodes in Season 1	Description
The Resident	Fox	Drama	Jan 2018	14	Focuses on the lives and duties of surgery, internal medicine and administrative staff in an Atlanta hospital with a specific interest in the bureaucratic practices of a hospital
The Good Doctor	ABC	Drama	Sept 2017	18	Highlights the trials and tribulations of a young autistic, savant surgical resident at a fictional San Jose hospital
Code Black	CBS	Drama	Sept 2015	18	Centered on four first-year residents and their colleagues who work in an overcrowded, understaffed and poorly funded ED in Los Angeles
Chicago Med	NBC	Drama	Nov 2015	18	Set in an urban ED, the show highlights how characters from a number of different training levels and specialties work together to provide state-of-the-art care
Grey’s Anatomy	ABC	Drama	March 2005	9	Follows the life of first-year surgical residents at a Seattle hospital as they struggle to balance a demanding residency schedule with drama in their personal lives
House	Fox	Drama	Nov 2004	22	Centered on a drug-addicted attending physician who often clashes with team members and hospital administration as he flouts rules and procedure to solve mystery ailments
Scrubs	NBC (later on ABC)	Comedy	Oct 2001	24	Told through the eyes of a first-year internal medicine resident, the series focuses on both the drama and humor of life as a first-year resident
ER	NBC	Drama	Sept 1994	25	Follows the lives, loves and losses of staff at a Chicago ED as they care for critically ill patients

The following data was recorded for each medical error: (1) role, race and gender of up to two healthcare providers responsible for the medical error; (2) race, gender and age of the victim of the medical error; (3) type of medical error; (4) disability experienced by the victim; (5) setting where the error occurred and (6) reporting of the medical error to the victim and subsequent action. Race and ethnicity were combined into one category referred to as “race” and categories were based on those laid forth by the US Census (White, Black or African American, Asian, American Indian or Alaskan Native, Native Hawaiian or other Pacific Islander and Hispanic) [15]. Age ranges for the victims of medical error were based on ranges (0-15, 16-44, 45-64, >65 years) used in the Harvard Medical Practice Study (henceforth, Harvard study) [3,4]. Types of medical error (operative, anesthesia, medical procedure, child birth, drug-related, improper diagnosis, improper therapy, falls and other) were based on categories used in both the Harvard study and Thomas et al.'s [5] study of medical centers in Utah and Colorado (henceforth, Utah/Colorado study) (Table 2). These represent two of the largest medical error studies conducted in the United States [3-5].

**Table 2:** Types of medical errors Categories and descriptions are based on the Harvard Medical Practice Study and Thomas et al.'s study of medical centers in Utah and Colorado.

Type of Error	Description
Operative	Issues arising up to two weeks after a surgical procedure that are the direct result of improper decision making by the surgical team
Anesthesia	Errors occurring during the administration, maintenance or withdrawal of both local and systemic anesthesia
Medical procedure	Errors occurring during a procedure done by a non-surgical team member in some location other than an operating room
Child birth	Errors related to labor, delivery or the post-partum period that result in harm to the mother or child
Drug related	Errors in administering the wrong type or dosage of medication; also includes improper prescription of controlled substances
Improper diagnosis	Patient was either given the incorrect diagnosis or there is a prolonged delay in determining the diagnosis that causes distress or additional impairment
Improper therapy	Patient is either given the wrong treatment or there is a prolonged delay in providing treatment, despite the proper diagnosis being concluded
Falls	Patient falls while under the supervision of hospital staff
Other	All other errors that do not properly fit into the described categories

Disability ratings for the victim (emotional trauma, insignificant injury, temporary injury, permanent minor/major and death) were also similar to those used in the Harvard and Utah/Colorado studies and were defined by the National Association of Insurance Commissioners (Table 3) [2-5]. These error and disability descriptions were provided to all reviewers to ensure reviewer consistency. Definitions were kept simple so that reviewer opinions could mirror those of the general public viewing these series. When the medical error resulted in physical injury, the type of injury was recorded using the following categories: laceration, bruise, rash, infection, fracture, burns, blood/fluid loss, loss of body part/organ, brain damage, thrombotic event, cardiac arrhythmia, seizure or other. The setting where the medical error occurred was categorized into inpatient care unit, emergency department, operating room (OR), labor and delivery, outpatient clinic, pharmacy, laboratory or other. Reporting of the medical error was categorized as error not reported to the victim, error reported without legal action taken or error reported with legal action taken. Reporting of the medical error needed to be performed before the end of the episode to be included in the analysis.

**Table 3:** Descriptions of disability categories Categories and descriptions are based on those provided by the National Association of Insurance Commissioners.

Disability Category	Description
Emotional trauma	The patient experiences an unnecessary, unpleasant emotion such as fear, sadness, etc., despite there being no physical harm
Insignificant injury	Lacerations, contusions, minor scars, rash, etc., where no delay in recovery occurs
Temporary injury	Infection, drug side effect, fracture set improperly, patient falls, etc., where recovery is complete but delayed
Permanent minor	Irreversible loss of fingers, loss or damage to organs, etc., where the injury is not disabling
Permanent major	Irreversible paraplegia, blindness, deafness, loss of limbs, brain damage, etc., which results in permanent disability
Death	Patient dies as a direct result of the medical error

Statistical analysis

For each area of interest (demographics, error type, disability, etc.) the percentage of each category was calculated for each series, as well as the summation of all eight TV series.

The average rate per hour was estimated using repeated measures Poisson regression for overall medical error, victim race, victim gender, type of medical error, setting of error and resultant disability, with adjustment for reviewer. This approach takes into account the varying duration of each show, along with the correlation among the multiple reviewers evaluating each series. Results were reported in terms of model-estimated average event rates per hour, 95% confidence intervals and p-values. The level of agreement among the four reviewers was assessed by evaluating the extended Kappa coefficient for measuring agreement among multiple raters, and reported with 95% confidence interval. Significance was defined as p<0.05.

Lastly, these findings were compared to real-life event rates from studies of US hospitals [3-5,8]. For areas where comparative studies utilized different categories, a qualitative comparison was given. For areas where studies with similar categories were present, a two-sided test of binomial proportions was used to assess statistical significance versus the US hospital proportion. If two studies were available for comparison, the average of these two studies was used. For setting of error and level of disability, our categories did not match perfectly with categories used in outside studies; therefore, adjustments were made. For setting, the category “inpatient room” included patient’s room and the ICU; the category “operating room” included OR, catherization lab and procedure rooms; the category “outpatient” included physician’s office, ambulatory care unit and day surgery, the category “other” included radiology and nursing homes. For disability, the temporary minor and temporary major categories were combined into one category of temporary injury.

## Results

Rate of medical error

The average rate of medical errors depicted in our sample of medical TV shows was 6.4 events per hour (95% CI 5.18-7.97). The rate of medical errors was significantly different among the eight series, ranging from 2.3/hr in ER to 9.5/hr in Code Black (p<0.001). Series released before 2005 had a rate of 7.20/hr (95% CI 6.34-8.06) whereas series released after 2005 had 5.54/hr (95% CI 4.33-6.75), which was not significantly different (p=0.35). There was a significant difference among reviewers in the average number of errors reported per hour (p<0.001), ranging from 2.5/hr to 14.8/hr. This variation was accounted for in all further statistical models. The extended Kappa coefficient was 0.20 (95% CI 0.08-0.32), indicating only low-moderate agreement among the four reviewers.

Demographics for the healthcare provider responsible for medical error

A White (75.6%), male (69.0%), attending physician (55.8%) was the most common provider responsible for an error (Table 4). Of note, “others”, which included physicians’ assistants, nurse practitioners, nurses, and other hospital staff, composed only 5.8% of the healthcare providers depicted as responsible. Additionally, despite several of the TV series in our sample focusing on an incoming intern class, Grey’s Anatomy and Scrubs were the only series that depicted interns as responsible for the majority of medical errors (70.0% and 80.0%, respectively). Although some studies categorize the healthcare provider responsible for the medical error by specialty of provider, no data from US hospitals addresses the role, race and gender for these providers.

**Table 4:** Demographic information for the healthcare provider responsible for the error including role, race and gender ^a^Includes physicians’ assistants, nurse practitioners, nurses, aids and other hospital staff. ^b^Healthcare provider responsible for the error was not shown on screen; therefore, sex and race could not be identified. ^c^Percentages add up to >100% in a given category because some errors had more than one person responsible for the error. *Denotes the most common healthcare provider responsible for the error, race and gender for each series, as well as overall.

		Role					Race					Gender		
Series	Errors	Attending	Fellow	Resident	Intern	Other^a^	White	Black	Asian	Hispanic	Other^b^	Male	Female	N/A^b^
The Resident	41	29	0	8	4	4	33	8	4	0	0	28	17	0
		(70.7%)*	(0.0%)	(19.5%)	(9.8%)	(9.8%)	(80.5%)*	(19.5%)	(9.8%)	(0.0%)	(0.0%)	(68.3%)*	(41.5%)	(0.0%)
The Good Doctor	23	10	0	7	7	2	15	6	1	4	0	20	6	0
		(43.5%)*	(0.0%)	(30.4%)	(30.4%)	(8.7%)	(65.2%)*	(26.1%)	(4.3%)	(17.4%)	(0.0%)	(87.0%)*	(26.1%)	(0.0%)
Code Black	50	26	9	3	18	0	35	14	7	0	0	35	21	0
		(52.0%)*	(18.0%)	(6.0%)	(36.0%)	(0.0%)	(70.0%)*	(28.0%)	(14.0%)	(0.0%)	(0.0%)	(70.0%)*	(42.0%)	(0.0%)
Chicago Med	31	17	8	5	1	0	25	2	4	0	0	15	16	0
		(54.8%)*	(25.8%)	(16.1%)	(3.2%)	(0.0%)	(80.6%)*	(6.5%)	(12.9%)	(0.0%)	(0.0%)	(48.4%)	(51.6%)*	(0.0%)
Grey’s Anatomy	30	8	2	0	21	2	24	2	5	2	0	20	13	0
		(26.7%)	(6.7%)	(0.0%)	(70.0%)*	(6.7%)	(80.0%)*	(6.7%)	(16.7%)	(6.7%)	(0.0%)	(66.7%)*	(43.3%)	(0.0%)
House	41	37	12	0	0	4	34	11	0	0	8	31	14	8
		(90.2%)*	(29.3%)	(0.0%)	(0.0%)	(9.8%)	(82.9%)*	(26.8%)	(0.0%)	(0.0%)	(19.5%)	(75.6%)*	(34.1%)	(19.5%)
Scrubs	15	3	0	0	12	0	8	5	2	0	0	11	4	0
		(20.0%)	(0.0%)	(0.0%)	(80.0%)*	(0.0%)	(53.3%)*	(33.3%)	(13.3%)	(0.0%)	(0.0%)	(73.3%)*	(26.7%)	(0.0%)
ER	11	5	0	4	0	2	9	0	0	0	2	7	2	2
		(45.5%)*	(0.0%)	(36.4%)	(0.0%)	(18.2%)	(81.8%)*	(0.0%)	(0.0%)	(0.0%)	(18.2%)	(63.6%)*	(18.2%)	(18.2%)
Overall^c^	242	135	31	27	63	14	183	48	23	6	10	167	93	10
		(55.8%)*	(12.8%)	(11.2%)	(26.0%)	(5.8%)	(75.6%)*	(19.8%)	(9.5%)	(2.5%)	(4.1%)	(69.0%)*	(38.4%)	(4.1%)

Demographics for the victim of medical error

The most common depicted victim was a White (73.6%) male (55.0%) between the ages of 16 and 44 (50.8%) (Table 5). Although the rate of medical error was significantly different among the victim’s race ranging from 0.7/hr for Hispanic to 5.0/hr for White (p<0.001), there was no significant difference among gender categories (2.7/hr for females, 3.9/hr for males, and 2.1/hr for unknown, p=0.19).

**Table 5:** Demographic Information for the victim of medical error including race, gender and age ^a^Patient could not be seen enough to determine the race, gender or approximate age (e.g., covered by drapes and then not discussed outside of that event). ^*^Denotes the most common victim race, gender and age for each series, as well as overall.

		Race					Gender			Age (Years)					Average Age
Series	Errors	White	Black	Asian	Hispanic	N/A^a^	Male	Female	N/A^a^	0-15	16-44	45-64	>65	N/A^a^	
The Resident	41	31	2	0	3	5	24	13	4	1	21	15	0	4	36.6
		(75.6%)^*^	(4.9%)	(0.0%)	(7.3%)	(12.2%)	(58.5%)^*^	(31.7%)	(9.8%)	(2.4%)	(51.2%)^*^	(36.6%)	(0.0%)	(9.8%)	
The Good Doctor	23	22	1	0	0	0	14	9	0	7	12	4	0	0	26.3
		(95.7%)^*^	(4.3%)	(0.0%)	(0.0%)	(0.0%)	(60.9%)^*^	(39.1%)	(0.0%)	(30.4%)	(52.2%)^*^	(17.4%)	(0.0%)	(0.0%)	
Code Black	50	24	23	0	3	0	20	29	1	4	27	17	1	1	38.4
		(48.0%)^*^	(48.0%)	(0.0%)	(6.0%)	(0.0%)	(40.0%)	(58.0%)^*^	(2.0%)	(8.0%)	(54.0%)^*^	(34.0%)	(2.0%)	(2.0%)	
Chicago Med	31	20	11	0	0	0	13	18	0	3	19	5	4	0	39.4
		(64.5%)^*^	(35.5%)	(0.0%)	(0.0%)	(0.0%)	(41.9%)	(58.1%)^*^	(0.0%)	(9.7%)	(61.3%)^*^	(16.1%)	(12.9%)	(0.0%)	
Grey’s Anatomy	30	25	2	0	0	3	15	13	2	10	7	5	4	4	30.3
		(83.3%)^*^	(6.7%)	(0.0%)	(0.0%)	(10.0%)	(50.0%)^*^	(43.3%)	(6.7%)	(33.3%)^*^	(23.3%)	(16.7%)	(13.3%)	(13.3%)	
House	41	37	0	4	0	0	31	10	0	0	33	8	0	0	29.3
		(90.2%)^*^	(0.0%)	(9.8%)	(0.0%)	(0.0%)	(75.6%)^*^	(24.4%)	(0.0%)	(0.0%)	(80.5%)^*^	(19.5%)	(0.0%)	(0.0%)	
Scrubs	15	12	0	0	0	3	9	6	0	0	4	8	0	3	46.7
		(80.0%)^*^	(0.0%)	(0.0%)	(0.0%)	(20.0%)	(60.0%)^*^	(40.0%)	(0.0%)	(0.0%)	(26.7%)	(53.3%)^*^	(0.0%)	(20.0%)	
ER	11	7	2	0	0	2	7	4	0	2	0	4	3	2	53.8
		(63.6%)^*^	(18.2%)	(0.0%)	(0.0%)	(18.2%)	(63.6%)^*^	(36.4%)	(0.0%)	(18.2%)	(0.0%)	(36.4%)^*^	(27.3%)	(18.2%)	
Overall	242	178	41	4	6	13	133	102	7	27	123	66	12	14	37.6
		(73.6%)^*^	(16.9%)	(1.7%)	(2.5%)	(5.4%)	(55.0%)^*^	(42.1%)	(2.9%)	(11.2%)	(50.8%)^*^	(27.3%)	(5.0%)	(5.8%)	

Our findings are consistent with an observational study that found that White males were the most common victims of medical error. However, whereas our data reported 74% of the victims were White, that observational study reported more diversity of victims with 46% White and 43% Black [8]. Code Black and Chicago Med, both focusing on urban EDs, were the only series to represent a close split between White and Black victims.

In terms of age, our data is inconsistent with US hospital data. Whereas the Harvard Study found that the majority of victims (40.6%) were >65 years of age, our data shows that only 5.8% of medical errors occurred in victims >65 years old [3]. Additionally, victims >65 years of age are only represented in four of the eight series.

Type of medical error

The most common types of medical error depicted were improper diagnosis (28.9%), operative error (19.4%) and drug-related error (12.0%) (Table 6). The rate of medical errors by type was significantly different (p<0.001), ranging from 1.1/hr for improper therapy to 2.7/hr for improper diagnosis. Series that focused on surgical residents, such as The Good Doctor and Grey’s Anatomy, had higher rates of improper diagnosis (48.0% and 29.0%, respectively) than surgical error (20.0% and 16.1%, respectively), whereas, ED-based series, such as Code Black and Chicago Med, had higher rates of operative error (38.0% and 38.7%, respectively) than improper diagnosis (14.0% and 6.5%, respectively).

**Table 6:** Types of errors represented in medical TV series Errors are expressed as percentage of total errors and compared with statistics from the Harvard and Utah/Colorado studies. Rate per hour is also provided. ^a^Includes unauthorized procedures/treatment/tests, administrative errors, falsifying labs/results and practicing under the influence of drugs/alcohol. ^b^Percentages may add up to >100% because some errors fit into more than one category. ^*^Denotes most common type of error in each series, as well as overall. ^ᶲ^Represents values that are significantly different from values reported in the Harvard and Utah studies.

Series	Errors	Operative	Anesthesia	Medical Procedure	Child Birth	Drug Related	Improper Diagnosis	Improper Therapy	Falls	Other^a^
The Resident	41	3	3	3	0	7	10	8	0	11
		(7.3%)	(7.3%)	(7.3%)	(0.0%)	(17.1%)	(24.4%)	(19.5%)	(0.0%)	(26.8%)^*^
The Good Doctor	23	5	0	2	0	2	12	3	0	1
		(21.7%)	(0.0%)	(8.7%)	(0.0%)	(8.7%)	(52.2%)^*^	(13.0%)	(0.0%)	(4.3%)
Code Black	50	19	7	6	4	3	7	3	0	1
		(38.0%)^*^	(14.0%)	(12.0%)	(8.0%)	(6.0%)	(14.0%)	(6.0%)	(0.0%)	(2.0%)
Chicago Med	31	12	5	5	3	0	2	0	0	4
		(38.7%)^*^	(16.1%)	(16.1%)	(9.7%)	(0.0%)	(6.5%)^*^	(0.0%)	(0.0%)	(12.9%)
Grey’s Anatomy	30	5	4	2	0	2	9	4	0	5
		(16.7%)	(13.3%)	(6.7%)	(0.0%)	(6.7%)	(30.0%)^*^	(13.3%)	(0.0%)	(16.7%)
House	41	0	0	4	0	13	19	5	0	2
		(0.0%)	(0.0%)	(9.8%)	(0.0%)	(31.7%)	(46.3%)^*^	(12.2%)	(0.0%)	(4.9%)
Scrubs	15	3	0	4	0	2	3	1	0	2
		(20.0%)	(0.0%)	(26.7%)^*^	(0.0%)	(13.3%)	(20.0%)	(6.7%)	(0.0%)	(13.3%)
ER	11	0	0	0	0	0	8	3	0	0
		(0.0%)	(0.0%)	(0.0%)	(0.0%)	(0.0%)	(72.7%)^*^	(27.3%)	(0.0%)	(0.0%)
Overall^b^	242	47	19	26	7	29	70	27	0	26
		(19.4%)ᶲ	(7.9%)ᶲ	(10.7%)	(2.9%)	(12.0%)ᶲ	(28.9%)^*,^ᶲ	(11.2%)ᶲ	(0.0%)	(10.7%)ᶲ
US hospital data		48.9%	1.2%	10.6%	2.6%	17.5%	6.9%	4.8%	1.5%	5.83%
Rate per hour		2.6	1.8	1.2	1.5	1.9	2.7	1.1	0.0	1.9

Our findings are inconsistent with US hospital data where operative errors are the most common medical error [4,5]. Of note, improper diagnosis was significantly more common in our sample compared with US hospital statistics (28.9% vs. 6.9%, respectively, p<0.001) and operative error was significantly less common (19.4% vs. 48.8%, respectively, p<0.001) [4,5]. Other types of errors that varied significantly between TV and US hospital statistics are denoted by phi (ᶲ) in Table 6.

Setting where the error occurred

The most common settings where errors occurred were on inpatient care units (34.7%) and the ED (31.4%) (Table 7). The rate of errors per setting was significantly different (p<0.001), with the highest rate occurring in inpatient care units (3.6/hr) and the lowest rate occurring in outpatient clinics (1.2/hr). In general, where the majority of errors occurred varied with the main setting of each series. The only exception to this trend is The Good Doctor, which focuses on surgical residents but had the majority of errors occur in the ED (39.1%).

**Table 7:** Setting where the error occurred in medical TV series Data is expressed as percentage of total errors and compared with statistics from the Harvard and Utah/Colorado studies. Rate per hour is also provided. ^a^Includes ambulances, site of an emergency, radiology, nursing homes or unknown locations. ^*^Denotes the most common location for each series, as well as overall. ^ᶲ^Represents values that are significantly different from values reported in the Harvard and Utah studies.

Series	Errors	Inpatient Care Unit	Emergency Department	Operating Room	Labor & Delivery	Outpatient Clinic	Pharmacy	Lab	Other^a^
The Resident	41	11	6	10	0	8	0	4	2
		(26.8%)*	(14.6%)	(24.4%)	(0.0%)	(19.5%)	(0.0%)	(9.8%)	(4.9%)
The Good Doctor	23	5	9	6	0	0	0	0	3
		(21.7%)	(39.1%)*	(26.1%)	(0.0%)	(0.0%)	(0.0%)	(0.0%)	(13.0%)
Code Black	50	21	21	4	0	1	0	0	3
		(42.0%)	(42.0%)*	(8.0%)	(0.0%)	(2.0%)	(0.0%)	(0.0%)	(6.0%)
Chicago Med	31	6	21	3	0	0	0	0	1
		(19.4%)	(67.7%)*	(9.7%)	(0.0%)	(0.0%)	(0.0%)	(0.0%)	(3.2%)
Grey’s Anatomy	30	8	6	11	0	0	0	2	3
		(26.7%)	(20.0%)	(36.7%)*	(0.0%)	(0.0%)	(0.0%)	(6.7%)	(10.0%)
House	41	23	6	0	0	5	3	0	4
		(56.1%)*	(14.6%)	(0.0%)	(0.0%)	(12.2%)	(7.3%)	(0.0%)	(9.8%)
Scrubs	15	10	0	4	0	0	0	1	0
		(66.7%)*	(0.0%)	(26.7%)	(0.0%)	(0.0%)	(0.0%)	(6.7%)	(0.0%)
ER	11	0	7	0	0	2	0	0	2
		(0.0%)	(63.6%)*	(0.0%)	(0.0%)	(18.2%)	(0.0%)	(0.0%)	(18.2%)
Overall	242	84	76	38	0	16	3	7	18
		(34.7%)*,ᶲ	(31.4%)ᶲ	(15.7%)ᶲ	(0.0%)	(6.6%)	(1.2%)	(2.9%)	(7.4%)
US hospital data		25.2%	3.14%	47.8%	6.1%	8.8%	N/A	N/A	8.7%
Rate per hour		3.6	2.7	1.9	0.0	1.2	1.42		0.7

Our findings differ from US hospital data, where the majority of errors occur in the OR [4,5]. TV shows in our sample had significantly more errors occur in the ED (31.4% vs. 3.14%, respectively, p<0.001) and care unit (34.7% vs. 25.3%, respectively, p<0.001) and significantly fewer errors occur in the OR (15.7% vs 47.9%, respectively, p<0.001) when compared to US hospital data [4,5]. It could not be determined if there was a significant difference between errors that occurred in the pharmacy and laboratory because these settings were not included in outside studies.

Level of disability for the victim

The most common levels of disability were emotional trauma (37.6%) and temporary injuries (30.2%) (Table 8). The rate of disability type varied significantly (p<0.001), ranging from 0.9/hr for permanent disability to 3.6/hr for emotional trauma. The most common types of bodily injury were cardiac arrest/arrhythmia (22.3%), blood loss (11.4%) and infection (7.4%). In general, more severe levels of disability, such as permanent disabilities and death, were not highly represented. Of note, The Resident was the only series with a high number of medical errors resulting in victim death (26.8%).

**Table 8:** Level of victim disability represented in medical TV series Data is expressed as percentage of total errors and compared with statistics from the Harvard and Utah/Colorado studies. Rate per hour is also provided. ^a^Patient outcome after the error is not discussed. ^*^Denotes the most common level of disability for each series, as well as overall. ^ᶲ^Represents values that are significantly different from values reported in the Harvard and Utah studies.

Series	Errors	Emotional trauma	Insignificant injury	Temporary Injury	Permanent minor	Permanent major	Death	Unknown^a^
The Resident	41	14	4	7	3	2	11	0
		(34.1%)^*^	(9.8%)	(17.1%)	(7.3%)	(4.9%)	(26.8%)	(0.0%)
The Good Doctor	23	10	2	9	0	0	1	1
		(43.5%)^*^	(8.7%)	(39.1%)	(0.0%)	(0.0%)	(4.3%)	(4.3%)
Code Black	50	15	15	19	1	0	0	0
		(30.0%)^*^	(30.0%)^*^	(38.0%)	(2.0%)	(0.0%)	(0.0%)	(0.0%)
Chicago Med	31	19	6	4	0	1	1	0
		(61.3%)^*^	(19.4%)	(12.9%)	(0.0%)	(3.2%)	(3.2%)	(0.0%)
Grey’s Anatomy	30	7	9	4	2	1	3	4
		(23.3%)	(30.0%)^*^	(13.3%)	(6.7%)	(3.3%)	(10.0%)	(13.3%)
House	41	16	4	19	0	2	0	0
		(39.0%)^*^	(9.8%)	(46.3%)	(0.0%)	(4.9%)	(0.0%)	(0.0%)
Scrubs	15	8	0	6	0	0	1	0
		(53.3%)^*^	(0.0%)	(40.0%)	(0.0%)	(0.0%)	(6.7%)	(0.0%)
ER	11	2	0	5	0	0	2	2
		(18.2%)	(0.0%)	(45.4%)^*^	(0.0%)	(0.0%)	(0.0%)	(0.0%)
Overall	242	91	40	73	6	6	19	7
		(37.6%)*,^ᶲ^	(16.5%)^ᶲ^	(30.2%)^ᶲ^	(2.5%)	(2.5%)	(7.9%)	(2.9%)
US hospital data		N/A	33.8%	45.1%	4.5%	2.9%	10.1%	3.3%
Rate per hour		3.6	2.1	2.3	0.93		1.7	1.6

Our findings differ from US hospital data. The Harvard study found the majority (56.8%) of medical errors result in insignificant injury, and the Utah/Colorado study found the majority (73.8%) of medical errors result in a temporary minor disability [3,5]. TV series showed fewer errors that resulted in insignificant injuries (16.5 vs. 33.8%, respectively, p<0.001) and temporary injuries (30.2% vs. 45.2%, respectively, p<0.001) [3,5]. Although exact numbers were unavailable, <1% of errors were estimated to result in only emotional injury whereas it represented 37.6% of errors in our sample [5].

Medical error reporting

The majority of medical errors depicted in our sample were not reported to the victim (49.2%), and only 3.7% of errors resulted in legal action (Table 9). The only series that depicted the majority of medical errors being reported to the victim were The Good Doctor and House. Additionally, only The Resident, The Good Doctor and ER depicted any legal action being taken against the provider responsible for the medical error.

**Table 9:** Reporting of medical errors ^*^Denotes more common reporting outcome for each series as well as TV shows overall.

Series	Errors	Error Not Reported to the Victim	Error Reported to the Victim, but No Legal Action Taken	Error Reported to the Victim and Legal Action Taken Against the Provider
The Resident	41	23	12	6
		(56.1%)^*^	(29.3%)	(14.6%)
The Good Doctor	23	8	14	1
		(34.8%)	(60.9%)^*^	(4.3%)
Code Black	50	31	19	0
		(62.0%)^*^	(38.0%)	(0.0%)
Chicago Med	31	18	13	0
		(58.1%)^*^	(41.9%)	(0.0%)
Grey’s Anatomy	30	15	15	0
		(50.0%)^*^	(50.0%)	(0.0%)
House	41	11	30	0
		(26.8%)	(73.2%)^*^	(0.0%)
Scrubs	15	8	7	0
		(53.3%)^*^	(46.7%)	(0.0%)
ER	11	5	4	2
		(45.5%)^*^	(36.4%)	(18.2%)
Overall	242	119	114	9
		(49.2%)^*^	(47.1%)	(3.7%)

Although there are no studies for direct comparison, prior studies have reported that as many as 85%-90% of medical errors may go unreported each year [16]. Therefore, medical error reporting may occur more often in TV series than real life.

## Discussion

To our knowledge, this is the first study to look at the depiction of medical errors in a sample of medical TV shows, allowing for a comparison between dramatized shows and reality. We were able to examine several medical series over a broad range of years, settings, and genres to get a diverse sample of medically based TV programming. In order to ensure consistency and objectivity, four medical students reviewers were provided pre-defined definitions and categories for all data points. The use of categories previously described in the medical error literature allowed for direct comparisons between TV shows and reality. Following collection and analysis, we have highlighted several discrepancies in the depiction of medical errors compared with statistics from US hospitals that could affect medical consumers in a variety of ways.

The dramatization of medical errors in medical TV series may lead to an unnecessary amount of fear and anxiety in its viewers. As discussed above, TV series depicted medical errors as resulting in mostly emotional trauma or temporary injury. Although most patients experienced only emotional trauma, negativity bias argues that viewers are more likely to remember and have their opinions formed by negative experiences than positive [17]. Leveraging this theory, patients are more likely to recall the negative outcomes depicted on TV, thus increasing their anxiety surrounding medical care. Additionally, medical TV series overrepresent certain types of errors such as improper diagnosis and improper therapy while underrepresenting operative error. This could lead to patients experiencing more anxiety during the diagnostic stage, despite there being a lower risk for error [4-5].

The increased fear and anxiety experienced by consumers of medical TV shows may lead to an increased number of medicolegal cases. As discussed above, the most common types of medical errors depicted on TV were improper diagnosis or operative error, resulting in emotional trauma or temporary injury. Viewers of medical TV shows may react to depictions of these types of errors by feeling empowered to advocate for similar errors they experienced. Fitting with this theory, although the most common medical error is an operative error, the most commonly filed medical malpractice claim is an error in diagnosis [4,5,18]. However, the poor reporting of medical errors depicted by TV series may lead to excessive anger and revenge that results in an increased number of unnecessary medicolegal cases. For example, although 7.4% of physicians have a malpractice claim filed against them each year, only 22% of these claims hold up in a court of law and result in compensation [18,19].

These dramatizations might also affect the patient-physician relationship that can result in poor healthcare utilization and health outcomes. Based on our sample, the most common healthcare provider depicted as responsible for an error was an attending physician. The attending physician should represent the healthcare provider with the most knowledge and expertise, overseeing physicians in training, other specialty physicians, advanced practice clinicians and other staff. By watching medical errors and adverse events occur on TV, viewers may begin to mistrust their attending physician and develop a negative view of the entire healthcare system. Patients with lower levels of physician trust are not only more likely to delay seeking medical care, but also they have higher rates of treatment discordance, poor treatment follow-through and overall worse health outcomes [20-22].

The depiction of medical errors in medical TV shows may also impact the education and training of healthcare providers, as well as the development of hospital policies and procedures to ensure safety. After being exposed to the medical errors depicted on TV, viewers in the medical field may feel inspired to educate themselves and others on the prevention of errors and how to properly communicate with victims of error. For example, many medical schools are creating curricula that educate students on how to recognize medical errors and employ proper methods of error disclosure [23,24]. Some studies have even examined the use of medical TV shows such as House and Grey’s Anatomy to introduce the topics of ethics and teamwork [25]. For already practicing physicians, states are starting to require that some hours of continuing education be dedicated to the prevention and disclosure of medical error [26]. At the institutional level, many hospitals have implemented error prevention protocols based on the Swiss cheese model that centers around the types of errors heavily represented in our sample of TV shows [27]. For example, to prevent against operative and procedural errors, hospitals have enacted policies such as pre-procedure site marking, time-outs to confirm identity and procedure prior to incision, and equipment counts. To prevent against errors in diagnosis, hospitals have enacted policies such as simplification of the electronic medical record for the ease of accessing and interpreting results, providing diagnostic checklists or algorithms, and implementing computer programs that integrate patient information to suggest possible diagnoses [28].

This study is not without limitations. Although we selected eight medical TV series representing a broad range of years, settings, and genres, our results may not be generalizable due to exclusion of many other medical TV series. Despite only analyzing a subset of episodes from each series, we chose to include episodes from both the beginning and end of each season allowing for appreciation of the entire plot line. In addition, analyzing a certain number of episodes per season prevented series with more episodes from overshadowing the results of other series for the cumulative results. Second, we recognize that the diversity of races and ages depicted on TV does not always mirror the diversity of our nation and thus may skew the demographic data [29,30]. However, it is important to recognize these demographic differences and how they may affect viewer attitudes. Third, there was significant variability among reviewers in their rate of medical error reporting. We attempted to limit the amount of variability by providing standardized definitions and selecting medical students with similar levels of training; however, we acknowledge that each reviewer may have implicit biases that affects his/her interpretation of the depicted events. This variability was accounted for in our statistical models by adjusting for reviewer in each model. Despite this adjustment, the extended Kappa coefficient showed only low-moderate agreement among the four reviewers. We believe that this variability allows for appreciation of the variability that would exist among common viewers. Fourth, our results for the medical error rate are expressed as errors per hour rather than errors per patient admitted, and direct comparisons could not be made in regard to rate. We considered recording error as errors per patient depicted on a show, but felt this would falsely elevate the rate of medical error since not all patients in a TV hospital receive screen time and there is no way to estimate bed capacity of these fictional hospitals. Lastly, this study was only able to describe the frequency of medical error depicted on TV and provide comparisons to US hospital data. Although we have discussed how viewing the depiction of medical errors may affect viewers and healthcare provider attitudes, a correlation between the two was not measured.

## Conclusions

Based on our sample of medical TV shows, we found discrepancies in the depiction of medical errors compared with statistics from US hospitals. This may lead to excessive fear and anxiety in viewers that results in delays in seeking medical care and increased medicolegal cases. Medical schools and healthcare systems are already instituting methods to prevent medical errors and educate providers on methods of proper error disclosure. Future studies should focus on determining whether exposure to medical TV shows affects the viewer attitudes towards healthcare providers and systems, as well as the outcomes of these attitudes such as delays in care or a higher number of medicolegal cases.
